# Association among race/color, gender, and intrinsic capacity: results from the ELSI-Brazil study

**DOI:** 10.11606/s1518-8787.2023057004548

**Published:** 2023-04-27

**Authors:** Jessica Plácido, Valeska Marinho, José Vinicius Ferreira, Ivan Abdalla Teixeira, Erico Castro Costa, Andrea Camaz Deslandes

**Affiliations:** I Universidade Federal do Rio de Janeiro Instituto de Psiquiatria Rio de Janeiro RJ Brasil Universidade Federal do Rio de Janeiro. Instituto de Psiquiatria. Rio de Janeiro, RJ, Brasil; II Fundação Oswaldo Cruz Instituto René Rachou Núcleo de Estudos em Saúde Pública e Envelhecimento Belo Horizonte MG Brasil Fundação Oswaldo Cruz. Instituto René Rachou. Núcleo de Estudos em Saúde Pública e Envelhecimento. Belo Horizonte, MG, Brasil

**Keywords:** Adult, Aging, Cognitive Dysfunction, Race Factors, Gender and Health

## Abstract

**OBJECTIVE:**

To investigate associations among race/color, gender, and intrinsic capacity (IC) (total and by domains) in middle-aged and older adults from a Brazilian cohort. As a secondary objective, we investigate these associations across Brazilian regions.

**METHODS:**

This is a cross-sectional study conducted with baseline data from the 2015–2016 Brazilian Longitudinal Study of Aging (ELSI-Brazil). IC was investigated via cognitive (verbal fluency), physical (gait velocity/handgrip), and psychosocial (Center for Epidemiological Studies Depression) domains. Moreover, IC sensory domain was evaluated via self-reported sensory disease diagnoses (vision and/or hearing impairment) and race/color was identified via self-reported criteria.

**RESULTS:**

We evaluated a total of 9,070 participants (aged ≥ 50 years). Black and Brown participants were 80% and 41% more likely to show a worse IC cognitive domain than white controls, respectively (OR = 1.80, 95%CI: 1.42–2.28, p < 0.001 and OR = 1.41, 95%CI: 1.21–1.65, p < 0.001). Moreover, Black and Brown women had almost a threefold greater chance of showing a worse IC than white men (OR = 2.91, 95%CI: 1.89–4.47, p < 0.001 and OR = 2.51, 95%CI: 2.09 - 3.02, p < 0.001) and a 62% (OR = 1.62, 95%CI: 1.02–2.57) and 32% (OR = 1.32, 95%CI: 1.10–1.57) greater risk of falling below our IC score cutoff point than white women. We found the greatest differences in the Brazilian South, whereas its North showed the lowest associations among race/color, gender, and IC.

**CONCLUSION:**

IC racial and gender disparities reinforce the need for public health policies to guarantee equality during aging. Promoting greater access to good health care requires understanding how racism and sexism can contribute to health inequities and their consequences in different Brazilian regions.

## INTRODUCTION

Many historically marginalized groups experience poorer health outcomes due to their limited access to healthcare, discrimination, and socioeconomic status, among other factors^1^. Black adults have a 50%, 26%, and 65% higher risk of hypertension, dyslipidemia, and dementia, respectively^2,3^. They also have higher odds of dying from cancer and kidney and cardiovascular disease than white ones^4^. Brazil has the largest number of Black individuals outside Africa and was the last country on the American continent to abolish slavery. About 55.8% of the Brazilian population self-reports as Black (8.6%) or Brown (46.8%)^5^. Currently, estimates speculate that 48% of its older population consists of self-reported Black (8.8%) or Brown (39.2%)^5^individuals. Black Brazilian older adults also show greater health vulnerability, risk of chronic diseases, cognitive impairment, and worse health status than white ones^4,6^. These results highlight the need to apply a racial perspective to the planning and monitoring of public health strategies.

Brazil is a continental country with significant sociodemographic differences across its regions. While its Southeast and South have the highest human development indices, its Northern and Northeastern states have the highest concentration of people earning up to half a salary per month^5^. These differences may extend to regional inequalities in health. A recent study showed that older adults who live in the North and Northeast have worse cognition than their Southern and Midwestern counterparts, showing the importance of observing the health status of Brazilian older adults across regions^7^.

The recent World Report on Ageing and Health, published by the World Health Organization, has changed its assessment to consider functional aging as healthy rather than viewing it from a disease perspective^8^. The report defines healthy aging as “the process of developing and maintaining the functional ability that enables well-being in older age.” It also points out that intrinsic capacity (IC) can determine functional ability, which can be monitored to provide earlier opportunities for intervention. IC constitutes the composite of individuals’ physical and mental capacities and comprises five key domains: cognitive; vitality; and psychosocial, sensory, and locomotor abilities, including gait, balance, and strength^8^.

Beard et al.^9^ (2019) have reported that IC scores are associated with clinical outcomes such as multimorbidity, cognitive decline, and dementia. It can also relate to a decline in basic activities of daily living, lower instrumental activities of daily living, and greater care dependence. In a prospective study, Zheng et al.^10^ (2018) have shown that older hospitalized patients with higher IC scores have a 62% lower mortality risk and a 24% lower chance of dependency after discharge. A recent review also highlights that socioeconomic factors, such as gender and income, influence IC^11^. It is essential to understand that the concept of IC was based on a longitudinal viewpoint that considers the quality of individuals’ interaction with environmental factors throughout their lives^11^. Thus, the consequences of social and health inequities can contribute to lower IC among some groups, such as women and historically marginalized populations. Although the literature still lacks an assessment of IC scores via an ethnic/racial perspective, when research considers IC domains separately, evidence shows that Black older adults have a greater risk of cognitive, gait decline, and depressive symptoms than white individuals^12^, poorer episodic memory, and the lowest handgrip scores^13,14^. Unfortunately, to the best of our knowledge, no study have assessed IC scores and sensory impairments via a race/color perspective.

It is essential to emphasize the influence of intersectionality (the multiplicative effect of inequalities experienced by nondominant marginalized groups) when observing the effect of social disparities^15^. Gayman and Barragan^16^(2012) have shown that individuals with two or more reasons for discrimination (e.g., Black women) were at 2.72 times higher risk for depressive symptoms than subjects with no social factors for discrimination (e.g., white, cisgender, and heterosexual men) and at 2.27 times higher risk than individuals with only one reason for discrimination (e.g., white women or Black men). Research also finds this effect in outcomes such as health self-perception and the prevalence of metabolic and cardiovascular diseases^17,18^. Thus, since failure to observe these intersections could lead to inaccurate diagnoses of health inequalities, it is essential to observe not only racial disparity but how race/color interacts with other types of oppression to increase health vulnerability.

Experiences of sexism and racism cause Black women to show worse sociodemographic data, such as lower educational attainment and income, in addition to a greater experience of loneliness and stress throughout their lives^19^. Moreover, cultural expectations of infinite resilience, independence, and strength commonly imposed on Black women (“the mask of the strong Black women”) increase the odds of this group showing physical and psychological distress^20^. Furthermore, studies have found that older Black women had worse cognitive and physical health than their white counterparts. In a longitudinal study, Garcia et al.^21^ (2019) showed that cognitively normal life expectancy (the proportion of individuals’ life span without cognitive impairment or dementia) for Black women at 50 years of age is 18.1 years, whereas it is about 27.9 years for white women. Black women also showed lower gait speed and more severe disabilities^22^. Unfortunately, despite results highlighting the importance of integrating both paradigms in health research, the literature still has a gap, especially regarding quantitative studies investigating the relation between race/color and gender with physical, psychological, and cognitive outcomes in older adults.

Moreover, existing studies assess race/color and gender as separate categories of analysis or, when examining them together, they view them as additive rather than mutually reinforcing and inseparable. This approach could introduce a risk of statistical bias, such as high collinearity between model variables, and potentially obscure disparities. We should highlight that most studies reported in the literature show data from North American and European populations. Thus, this study investigates the associations among race/color, gender, and IC in middle-aged and older adults from a Brazilian cohort. As a secondary objective, we investigate the possible existence of macro-regional variations in these associations.

## METHODS

This cross-sectional study was conducted with baseline data from the Brazilian Longitudinal Study of Aging (ELSI-Brazil), conducted between 2015 and 2016. ELSI-Brazil is a prospective cohort study conducted in a representative sample of non-institutionalized community-dwelling Brazilian individuals. To collect its data, participants aged 50 years and over were included and a multistage stratified cluster sampling design, adopted to select our study sample. Municipalities were divided into four strata according to their population size. Our sample was chosen in three stages: municipalities, census tract, and household. In the first three strata, which included municipalities with up to 750,000 inhabitants, the sample was selected in three consecutive stages: municipalities, census tracts, and households. Our sample was selected in a fourth strata, which included larger municipalities (> 750,000 inhabitants) in two stages: census tract and households. The Lavallée and Hidiroglou method was used to quantify the size and number of municipalities allocated to each stratum^23^. An inverse sampling design was used by ELSI-Brazil to avoid increasing its sample size to compensate for nonresponses and enable investigators to define how many units needed to be observed to obtain a prespecified number of successes. Moreover, sample weights were derived to account for differential selection probabilities and nonresponses. The details of the ELSI-Brazil methodology were described elsewhere^24^. For this study, middle-aged and older adults (> 50 years old) of all genders who self-identify as Black, Brown or white, according to the Brazilian Institute of Geography and Statistics (IBGE) criteria, were included^25^. IBGE investigates the color or race of the Brazilian population by their self-reports. Thus, people are asked about their color according to the following options: white, Indigenous, Yellow, Brown or Black. The last two compose the population racially known as Black. Our exclusion criteria consisted of the presence of dementia, cognitive impairment or Parkinson’s diagnosis and decline in the ability to perform daily living activities. Subjects who failed to perform any of the motors or cognitive tests composing IC scores were also excluded. Data referring to the population who declares itself Yellow and Indigenous were excluded due to their small size in our sample. A total of 9,070 individuals were evaluated and initially included in this study. However, 310 participants were excluded due to the presence of dementia or Parkinson’s disease (n = 146), impairments in activities of daily living (n = 100), and unconcluded evaluations (n = 64). Our final sample was composed of 5,170 Black (Black = 887, Brown = 4,283) and 3,590 white adults. Written consent forms were signed by all patients. This study was approved by the research ethics committee from the Oswaldo Cruz Foundation-Minas Gerais (CAAE: 34649814.3.0000.5091).

### Procedures and Tests

Households, individual participant interviews, physical measurements, laboratory tests, and blood aliquots for future analysis were included in our ELSI-Brazil baseline data collection. Moreover, the database was accessed via *www.elsi.cpqrr.fiocruz.br* and information on sociodemographic characteristics (age, educational attainment, gender, income, region, and health insurance), anthropometric measurements, health status, personal habits, health self-perception, perceived discrimination in medical appointments, and self-reported history of chronic and mental diseases diagnosed by a physician were collected for this study. To construct the IC scores, the five domains previously proposed by the WHO guideline were considered: cognitive, psychosocial, vitality, locomotor, and sensory domains.

### Intrinsic Capacity Domains

Intrinsic Capacity was evaluated by five domains: cognitive, psychosocial, vitality, locomotor, and sensory ones. The cognitive domain was assessed by the Verbal Fluency Test (VF) according to the cutoff points proposed by Brucki et al.^26^. This task was validated for Brazilian older adults with a cutoff point of 9 points for individuals with low educational attainment (less than eight years) and 13 for those with > 8 years of education. During the task, participants are asked to name as many animals as possible in one minute^26^. The psychosocial domain was evaluated by depressive symptoms via the eight-item Center for Epidemiological Studies Depression (CES-D) with a cutoff point of ≥ 4^27^. Vitality was based on handgrip strength, measured via a digital hand dynamometer. The test was applied three times and the highest mean peak value between both hands was then recorded for analysis. Cutoff values were classified by the Cardiovascular Health Study, which considers participants’ Body Mass Index and gender^28^. Lastly, gait velocity was assessed by a cutoff point of < 0.8 m/s, and the IC locomotor and sensory domains by the presence of visual or auditory impairments (diagnosed by a physician), respectively^29^. IC scores were created and calculated based on the presence or absence of impairment in each IC domain (total score: min = 0, max = 5) and individuals with a score below 3 were considered as having low IC.

### Covariates

Age (50–59, 60–69, and 70 years or older), gender (female and male), educational attainment (never studied, adult education, 1–4 years, 5–8 years, 9–11 years, and 12 years or more), and income (less than one minimum wage, 1–3 minimum wages, 4–6 minimum wages, 7–9 minimum wages, and more than 9 minimum wages) were used as covariates in this study. To guide the selection of possible adjustment variables, a causal diagram was built via the DAGitty software (Supplementary Figure 1). Results suggested two types of analysis: total effect (which dispenses with adjustments) and direct effect analysis, in which age, gender, educational attainment, and income variables must be controlled to assess the direct effect of race/color on IC. To control our analyses, individuals aged 50–59 years who earned less than one minimum wage, had never studied, and self-reported as women were used as a reference group.

**Figure 1:**
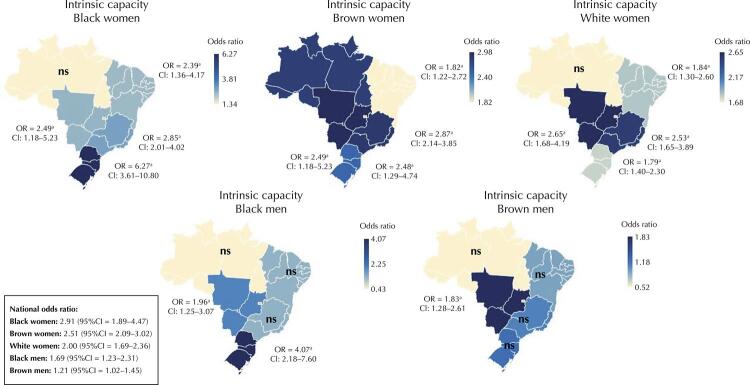
Association between race/color, gender, and IC (white men as a reference group) divided by Brazilian regions (Southeast, North, Northeast, Midwest, and South). The bidirectional heatmap shows higher (dark blue) and lower (beige) risk of a worst IC (white men as the reference group) ns: non-significant; OR: odds ratio; CI: confidence interval. Higher OR values mean worse IC levels than white men.

^a^ Statistical significance p < 0.05.

### Statistical Analysis

The chi-square test was used to compare categorical variables and a logistic regression was performed to evaluate associations among race/color, gender, and IC domains (cognitive, physical, locomotor, vitality, sensory, and psychological decline). To analyze the association between race/color and IC, white participants were used as reference, whereas to understand the relation among race/color, gender, and IC, white men were used as a reference group. Our sample was also divided by regions (Southeast, North, Northeast, Midwest, and South) to investigate the influence of macro-regional variations in those associations. The results of our regression analyses were controlled by age, gender, income, and educational attainment, except when these variables were already considered in the test cutoff points or in case of high collinearity inside the model. Statistical analysis was conducted via SPSS^®^, version 26.0 (IBM Corporation, New York, USA), and RStudio, version 1.3.1073. A p ≤ 0.05 was considered statistically significant. All analyses were carried out with procedures for complex samples, which include the sample weight of individuals and the sample design effect.

## RESULTS

Our three groups differed in age (*x*^2^= 35.7, p = 0.001), educational attainment (*x*^2^= 219.0, p < 0.001), and income (*x*^2^= 295.3, p < 0.001) and Black and Brown participants were younger and poorer than white ones. Black and Brown participants also had more than double the prevalence of illiteracy rates (20.6% and 15.3% versus 7.5%) (Table 1) and reported a worse health perception (*x*^2^= 61.8, p < 0.001), greater perceived discrimination during medical appointments (*x*^2^= 15.6, p = 0.008), and higher dependence on the public health system (*x*^2^= 116.0, p < 0.001). We found no difference in the proportion of men and women among our groups (*x*^2^= 2.33, p = 0.57) (Table 1). When we observed specific IC domains, Black and Brown participants performed worse on the VF test (*x*^2^= 57.8, p < 0.001) and showed more depressive symptoms (*x*^2^= 17.9, p = 0.009), whereas white volunteers had greater IC sensory impairment (*x*^2^= 87.7, p < 0.001). However, in general, Black and Brown participants showed a worse IC, with about 31.0% and 26.2% of our sample falling below cutoff points on the IC score, respectively (*x*^2^= 22.9, p = 0.01) (Table 1). Although the Brown group was younger (*x*^2^= 9.4, p = 0.03) and better educated (*x*^2^= 20.7, p = 0.01) than the Black group, we found no difference in IC levels (*x*^2^= 6.7, p = 0.12) between the two groups.

**Table 1:** Demographic, physical, and cognitive characteristics of Black, Brown, and white participants.

	Black participants (n = 887)	Brown participants (n = 4,283)	White participants (n = 3,590)	Total cohort (n = 8,760)	x^2^	p
Age (%)						
50–59	45.5	51.6	45.3	48.2	35.7	0.001^a^
60–69	33.8	28.7	30.2	29.9		
≥ 70	20.7	19.7	24.4	21.9		
Educational attainment (%)						
Never studied	20.6	15.3	7.5	12.4	219.0	< 0.001^a^
Adult education (years)	0.1	0.1	0.2	0.1		
1–4	40.5	38.1	36.9	37.8		
5–8	16.2	21.9	22.6	21.7		
9–11	17.0	18.5	20.4	19.2		
≥ 12	5.7	6.0	12.4	8.8		
Gender (%)						
Female	56.4	53.2	54.1	53.9	2.33	0.57
Male	43.6	46.8	45.9	46.1		
Income (%)						
Less than one minimum wage	78.5	75.0	57.6	67.6	295.3	< 0.001^b^
1–3 minimum wages	19.9	22.6	35.6	28.1		
3–6 minimum wages	1.0	2.2	4.5	3.1		
6–9 minimum wages	0.3	0.3	1.2	0.7		
More than 9 minimum wages	0	0.1	1.2	0.5		
Region (%)						
Southeast	48.0	42.1	50.4	46.4	975.2	< 0.001^b^
North	4.3	8.3	1.7	5.0		
Northeast	35.5	34.2	12.6	24.7		
Midwest	6.1	7.2	4.9	6.1		
South	6.1	8.2	30.3	17.8		
Verbal fluency (%)						
Below the cutoff point	36.0	30.2	23.6	27.8	57.8	< 0.001^b^
Above the cutoff point	64.0	69.8	76.4	72.2		
Depressive symptoms (%)						
Yes	31.9	30.7	26.3	28.9	17.9	0.009^b^
No	68.1	69.3	73.7	71.1		
Sensorial deficts (%)						
Yes	66.2	69.5	78.7	73.2	87.7	< 0.001^b^
No	33.8	30.5	21.3	26.8		
Handgrip strength (%)						
Below the cutoff point	25.2	26.8	24.4	25.6	4.47	0.35
Above the cutoff point	74.8	73.2	75.6	74.4		
Gait velocity (%)						
Below the cutoff point	42.8	33.7	32.2	33.9	26.5	0.01^b,c^
Above the cutoff point	57.2	66.3	67.8	66.1		
Intrinsic capacity score (%)						
Below the cutoff point	31.0	26.2	20.1	25.2	22.9	0.01^b^
Above the cutoff point	69.0	73.8	79.9	74.8		

x^2^: Pearson chi-square.

^a^ Significant difference (p ≤ 0.05) among white, Brown, and Black participants.

^b^ Significant difference (p ≤ 0.05) between Black and white participants.

^c^ Significant difference (p ≤ 0.05) between Brown and Black participants.

Our logistic regression analyses showed that Black and Brown adults had 80% and 41% more chance of having cognitive impairment (Black OR = 1.80, p < 0.001; Brown OR = 1.41, p < 0.001) than white ones. They also showed 54% and 24% more risk of worse IC (Black OR = 1.54, p = 0.004; Brown OR = 1.24, p = 0.002). Meanwhile, both groups had less risk of a diagnosis of visual and/or hearing impairment (Black OR = 0.60, p < 0.001; Brown OR = 0.70, p < 0.001). The sensory domain suffered the greatest influence of income; our results showed that individuals with more income had twice the chance of having received a diagnosis (OR = 2.16, p < 0.001), whereas Black ones were 61% more likely to have a worse gait speed (OR = 1.61, p < 0.001) and Brown people, 18% more likely to have worse handgrip strength (OR = 1.18, p = 0.02).

When we divided our data by race/color and gender, our results indicated that Black and Brown women are twice as likely to fall below the cutoff point in VF scores (Black OR = 2.28, p < 0.001; Brown OR = 1.86, p < 0.001) and on the CDS scale (Black OR = 2.40, p < 0.001; Brown OR = 2.32, p < 0.001). Moreover, they had almost three times more chance of showing a worse IC than white men (Black OR = 2.91, p < 0.001; Brown OR = 2.51, p < 0.001). Compared to white men, Black and Brown ones also performed worse in the cognitive (Black OR =1.81, p < 0.001; Brown OR = 1.36, p= 0.004), locomotor (Black OR = 2.13, p < 0.0001; Brown OR = 1.86, p < 0.0001), and vitality (Black OR = 1.52, p = 0.02; Brown OR =1.29, p = 0.01) domains and in IC scores (Black OR = 1.69, p < 0.0001; Brown OR = 1.21, p = 0.02). Results also point to a gender disparity between white men and women, in which women performed worse in all IC domains, especially in the psychosocial (OR = 2.0, p < 0.001) and sensory (OR = 2.43, p < 0.001) ones (Table 3). However, white women were 30% (OR = 0.70, 95%CI: 0.51–0.97) less likely to show a decline in the IC cognitive domain than Black men, suggesting the effect of intersectionality in these disparities. Lastly, Black and Brown women showed 62% (OR = 1.62, 95%CI 1.02–2.57) and 32% (OR = 1.32, 95%CI: 1.10–1.57) more risk of falling below the cutoff point in the IC score than white women, respectively.

**Table 3:** Total and direct effects of the associations among race/color, gender, and intrinsic capacity by each domain.

Cognition						
Verbal fluency	OR	95%CI	p	OR	95%CI	p
Black women	2.33	1.63–3.34	< 0.001^a^	2.28	1.60–3.23	< 0.001^a^
Brown women	1.88	1.52–2.31	< 0.001^a^	1.86	1.52–2.29	< 0.001^a^
White women	1.29	1.05–1.58	0.01^a^	1.26	1.03–1.55	0.02^a^
Black men	1.82	1.37–2.42	< 0.001^a^	1.81	1.37–2.40	< 0.001^a^
Brown men	1.35	1.09–1.67	0.006^a^	1.36	1.10–1.68	0.004^a^
Age				1.29	1.17–1.42	< 0.001^a^
Income				0.92	0.83–1.03	0.16
**Psychological**						
Depressive symptoms	OR	95%CI	p	OR	95%CI	p
Black women	2.60	1.99–3.38	< 0.001^a^	2.40	1.83–3.14	< 0.001^a^
Brown women	2.55	2.06–3.15	< 0.001^a^	2.32	1.88–2.86	< 0.001^a^
White women	1.97	1.63–2.38	< 0.001^a^	2.0	1.66–2.40	< 0.001^a^
Black men	1.26	0.94–1.69	0.11	1.12	0.83–1.51	0.43
Brown men	1.19	0.96–1.48	0.11	1.07	0.86–1.33	0.52
Age				0.78	0.73–0.84	< 0.001^a^
Income				0.69	0.61–0.78	< 0.001^a^
**Vitality**						
Handgrip	OR	95%CI	p	OR	95%CI	p
Black women	1.29	0.83–2.01	0.25	1.19	0.79–1.80	0.38
Brown women	1.76	1.41–2.19	< 0.001^a^	1.79	1.42–2.24	< 0.001^a^
White women	1.65	1.37–1.99	< 0.001^a^	1.60	1.32–1.95	< 0.001^a^
Black men	1.50	1.03–2.19	0.03^a^	1.52	1.05–2.20	0.02^a^
Brown men	1.25	1.01–1.55	0.03^a^	1.29	1.06–1.58	0.01^a^
Age				2.21	1.97–2.49	< 0.001^a^
Income				0.76	0.67–0.85	< 0.001^a^
**Locomotor**						
Gait velocity	OR	95%CI	p	OR	95%CI	p
Black women	3.04	2.07–4.46	< 0.001^a^	3.12	2.10–4.62	< 0.001^a^
Brown women	2.16	1.73–2.70	< 0.001^a^	2.27	1.81–2.85	< 0.001^a^
White women	2.12	1.73–2.60	< 0.001^a^	2.13	1.73–2.64	< 0.001^a^
Black men	1.77	1.27–2.47	< 0.001^a^	1.86	1.32–2.62	< 0.001^a^
Brown men	1.17	0.94–1.47	0.15	1.24	0.99–1.56	0.05
Age				2.03	1.84–2.23	< 0.001^a^
Income				0.86	0.76–0.97	0.01^a^
Vision/hearing impairments	OR	CI_95%_	p	OR	CI_95%_	p
Black women	0.44	0.33–0.55	< 0.001^a^	0.41	0.17–0.50	< 0.001^a^
Brown women	0.66	0.57–0.77	< 0.001^a^	0.77	0.67–0.90	< 0.001^a^
White women	2.26	1.87–2.73	< 0.001^a^	2.43	2.01–2.94	< 0.001^a^
Black men	0.57	0.44–0.74	< 0.001^a^	0.70	0.54–0.89	0.005^a^
Brown men	0.69	0.58–0.82	< 0.001^a^	0.80	0.67–0.95	0.01^a^
Age				0.94	0.86–1.04	0.26
Income				2.17	1.86–2.51	< 0.001^a^
**Intrinsic capacity**						
IC	OR	95%CI	p	OR	95%CI	p
Black women	3.31	2.12–5.17	< 0.001^a^	2.91	1.89–4.47	< 0.001^a^
Brown women	2.69	2.25–3.21	< 0.001^a^	2.51	2.09–3.02	0.01^a^
White women	2.04	1.73–2.40	< 0.001^a^	2.00	1.69–2.36	< 0.001^a^
Black men	1.87	1.37–2.55	< 0.001^a^	1.69	1.23–2.31	0.001^a^
Brown men	1.27	1.06–1.52	0.009^a^	1.21	1.02–1.45	0.02^a^
Age				1.67	1.52–1.83	< 0.001^a^
Income				0.76	0.69–0.83	< 0.001^a^
Education				0.87	0.83–0.92	< 0.001^a^

Reference group: white men who were aged 50–59 years, earned less than one minimum wage, and had never studied; OR: odds ratio; 95%CI: 95% confidence interval; IC: intrinsic capacity capacity score.

^a^ Statistical significance (p < 0.5).

Finally, when we categorized our participants by macroregions, we found the biggest disparities among South, Midwest, and Southeast. Southern Black women showed the worst IC scores (OR = 6.27, p < 0.001) and Midwestern Brown ones, nearly three times more risk of having the lowest IC levels (OR = 2.98, p < 0.001; Southeast OR = 2.87, p < 0.001) (Figure 1). However, by separately regarding the IC domains, the Brazilian South drew our attention for its high prevalence of depressive symptoms in Black men (OR = 3.79, p < 0.001) and Brown women (OR = 3.77, p < 0.001) and a worst cognitive performance in self-reported Black men than in white ones (OR = 5.00, p < 0.001), whereas the Northeast and Midwest stand out for their prevalence of worst cognitive performance in Black women (OR = 2.90, p < 0.001 and OR = 3.71 p < 0.001) (Supplementary Table 2).

**Table 2:** Total and direct effects of the associations among race/color and intrinsic capacity by each domain.

Cognition						
Verbal fluency	OR	95%CI	p	OR	95%CI	p
Black	1.82	1.43–2.32	< 0.001^a^	1.80	1.42–2.28	< 0.001^a^
Brown	1.40	1.20–1.63	< 0.001^a^	1.41	1.21–1.65	< 0.001^a^
Age				1.27	1.16–1.39	< 0.001^a^
Income				0.91	0.82–1.02	0.12
**Psychological**						
Depressive symptoms	OR	95%CI	p	OR	95%CI	p
Black	1.30	1.03–1.64	0.02^a^	1.17	0.93–1.47	0.17
Brown	1.23	1.05–1.46	0.01^a^	1.12	0.95–1.33	0.16
Age				0.78	0.73–0.84	< 0.001^a^
Income				0.69	0.61–0.78	< 0.001^a^
Gender				0.47	0.42–0.53	< 0.001^a^
**Vitality**						
Handgrip	OR	95%CI	p	OR	95%CI	p
Black	1.04	0.74–1.45	0.81	1.01	0.75–1.36	0.93
Brown	1.13	0.97–1.32	0.11	1.18	1.02–1.37	0.02^a^
Age				2.22	1.98–2.50	< 0.001^a^
Income				0.75	0.67–0.85	< 0.001^a^
**Locomotor**						
Gait velocity	OR	95%CI	p	OR	95%CI	p
Black	1.57	1.18–2.09	0.002^a^	1.61	1.20–2.15	0.001^a^
Brown	1.07	0.86–1.33	0.51	1.13	0.91–1.41	0.25
Age				2.02	1.84–2.23	< 0.001^a^
Income				0.86	0.76– 0.97	0.01^a^
Gender				0.51	0.44–0.59	< 0.001^a^
Sensorial						
Visual/hearing impairments	OR	95%CI	p	OR	95%CI	p
Black	0.53	0.41–0.67	< 0.001^a^	0.60	0.48–0.77	< 0.001^a^
Brown	0.61	0.52–0.72	< 0.001^a^	0.70	0.60–0.81	< 0.001^a^
Age				0.94	0.86–1.04	0.13
Income				2.16	1.85– 2.51	< 0.001^a^
Gender				0.50	0.44–0.56	< 0.001^a^
**Intrinsic capacity**						
IC	OR	95%CI	p	OR	95%CI	p
Black	1.72	1.23–2.40	0.001^a^	1.54	1.15–2.08	0.004^a^
Brown	1.28	1.11–1.48	< 0.001^a^	1.24	1.08–1.42	0.002^a^
Age				1.67	1.52–1.83	< 0.001^a^
Income				0.76	0.69–0.83	< 0.001^a^
Gender				0.50	0.43–0.57	< 0.001^a^
Education				0.87	0.83–0.92	< 0.001^a^

Reference group: white individuals who were aged 50–59 years, earned less than one minimum wage, had never studied, and self-reported as women; OR: odds ratio; 95%CI: 95% confidence interval; IC: intrinsic capacity a score.

^a^ Statistical significance p < 0.5.

## DISCUSSION

This study examined associations among race/color, gender, and IC in middle-aged and older adults. As a secondary objective, we explored the behavior of those associations among Brazilian regions. Impairment in most IC domains caused Black and Brown individuals to show the lowest IC scores. The magnitude of this disparity was most substantial when we divided our sample by race/color and gender, with Black and Brown women showing a higher risk of depressive symptoms and lower verbal fluency scores. However, we need to consider the regional differences in these disparities.

We also observed the impact of race/color and gender in the IC psychosocial domain, with Black and Brown women showing more depressive symptoms than white participants. These results agree with previous studies, which have reported that women had higher odds of depressive symptoms and major depression than men, especially if aged 65 years and older^30^. According to Liang et al.^31^, Black people are three times more likely to show moderate depressive symptoms than white individuals. These findings advance current knowledge by showing the mutual effect of gender and race on depressive symptoms in middle-aged and older adult populations. Still, when we look at these data, we should consider the gender and race/color issues involved in symptoms of depression. Men are usually underdiagnosed with depression even when they seek treatment, mainly because they show different symptoms than women, such as irritability, aggression, and substance abuse^32^. Building a “non-right to feel” can increase the chances of underdiagnosing anxiety and depression in this group^20^. Thus, research must build criteria and assessments which capture the nuances of race/color and gender in this population to obtain better diagnoses.

Despite their greater prevalence of depressive symptoms, Black adults received diagnosis of depression less frequently than white adults, indicating the possibility of misdiagnosis among this population. These findings resemble previous studies which have shown that Black individuals with depression, anxiety, and cancer have a higher chance of being underdiagnosed and undertreated than their white counterparts^33^. Moreover, according to Clark et al.^36^(2005), Black older adults take about seven years from the onset of their first symptoms of dementia to seek medical attention. These results are likely associated with barriers to accessing health care related to racism, such as Black individuals’ lower educational attainment, given that education could produce an understanding of the causes and symptoms of the disease and of the negative experiences of failing to seek help^37^. According to a recent report published by the Alzheimer’s Disease Association, 36% of Black Americans believe that discrimination would be a barrier to receiving Alzheimer’s care; they also claim to suffer more discrimination when seeking health services than white subjects (50% versus 9%)^38^. Thus, the planning of standardized IC composite scores should consider the effects of structural racism in choosing measurement tools which can identify impairment in each domain and capture IC scores in historically marginalized populations.

Regarding the cognitive domain of the IC score, Black and Brown adults showed worse semantic memory and executive function than white controls. Holtzer et al.^39^ (2020) have reported that, specifically in the Brazilian population, educational attainment, gender, and place of residence may influence performance on the verbal fluency task. In Brazil, Black individuals compose about 76% of its poor population and 32.9% of Black and Brown people live below the poverty line, configuring most *favela* residents. Concerning gender bias, our data show that Black or Brown women earn 44.4% and 58.6% of what white men and women do, respectively^5^. Regarding educational attainment, the Black and Brown population has three times more illiterate individuals than the white one^5^. This is particularly interesting when we look at the results in Salata^40^ (2020), which compared the direct, indirect, and total effects of race/color, social status, and income in an analysis based on data from the National Survey of Health collected in 2014. Their results showed that, although the total effects of social status are greater than those of race, social disparities are insufficient to explain most disadvantages Black Brazilian individuals experience, which, according to the author, occur mainly indirectly via education and occupational status.

Impairments in semantic and phonemic fluency tasks have been associated with increased odds of mild cognitive impairment and dementia diagnoses^41^. Although previous studies have shown a prevalence in Black individuals of genes such as apolipoprotein E (ApoE) ε4 — which could be associated with poorer cognitive performance —, evidence indicates that social factors, such as education and access to reading material, may more greatly contribute to these differences^16^. In a longitudinal study, Rajan et al.^41^ (2019) investigated cognitive resilience to ApoE ε4 in older Black and white adults during an 11-year follow-up study and reported that the strongest predictors of cognitive resilience among Black APOEε4 carriers were a higher literacy/educational level and the absence of diabetes mellitus. This result highlights the importance and role of early interventions promoting education and access to primary health care to increase cognitive reserve, even among individuals with a higher genetic risk.

Black adults also showed the worst outcomes in the IC locomotor domain, whereas Brown individuals, the lowest levels of handgrip strength. According to Ramírez-Vélez et al.^42^ (2019), older men above the cutoff point for muscle weakness, as measured by handgrip strength, had less risk for IC (38%), cognition (50%), and locomotor deficits (53%). In contrast, older women with higher handgrip strength scores showed lower odds of IC problems (21%), especially in its cognition (56%), locomotor (34%), and psychosocial (43%) domains. Moreover, we should highlight that gait performance has been one of the most studied outcomes in older populations and research has postulated that changes in gait velocity may indicate cognitive decline and dementia^43^.

The IC inequalities found in this study suffered regional influence. Although Black and Brown women in all regions showed worse results, some regions, especially the South, showed greater disparity, whereas the Brazilian North showed the lowest associations among race/color, gender, and IC. The South is inhabited mainly by white individuals (76.8% of the South declare themselves white, whereas 18.7%, Brown and only 3.8%, Black^5^) and is known for its population of European immigrants, especially Germans and Italians^5^. The Northern region has the highest poverty rates in the country, with an estimated average family income of around R$ 938.06 and the lowest rate of income inequality (2.7%). Moreover, the Brazilian North has one of the lowest human development indices and the highest rates of illiteracy (7.6% versus 3.3% in the South) and violent deaths than other Brazilian regions^5^.

In the context of this study, we can see that the IC differences associated with race/color are smaller for Brown men than Black ones, mainly in the North and Northeast. Likewise, despite its non-significance, Brown women have a better IC than those who declare themselves Black. We may explain these differences by these individuals’ better income and greater access to education and health services^5^. Effectively observing and understanding the differences between the Black and Brown populations in Brazil requires considering socio-historical processes, primarily those described by anthropologists, i.e., miscegenation and the myth of racial democracy^44,45^. After abolishing slavery, Brazil created policies to whiten its population. Thus, the concept of ‘Brown,’ rather than representing a race/ethnicity, refers to a biopolitical device of control used during the post-slavery period to whiten the Brazilian population, perpetrating its genocide against Black people and reinforcing the myth of racial democracy^44,45^. Moreover, the Brown concept aimed to deny racism and dissipate the possibility of racial conflicts like those in the USA in its post-slavery period^44,45^. Following this hypothesis, the differences between Black and Brown groups may be associated with issues involved in self-identification decisions, such as colonization processes^44,45^, regional identity construction^44,46^, and colorism^48^.

This study highlights the importance of including data on race/color and gender in public health policies and using an intersectional approach in health research. The Statute of Racial Equality^49^ and the Brazilian Ministry of Health consider the Black population to be the sum of self-reported Black and Brown individuals. The latter institution used this classification method to create the National Policy for the Integral Health of the Black Population^50^. Thus, future studies should independently observe the relation between color and race.

Moreover, our results reinforce the use of IC assessments as a low-cost tool to monitor older adults’ health status. Furthermore, note that Brazil has the highest population of Black individuals outside Africa and was the last country on the American continent to abolish slavery. Thus, promoting greater access to quality health care for all people worldwide requires understanding how race/color disparity can increase health inequities and their consequences in Brazil. However, we recognize that this study has limitations, such as measuring sensory parameters and diagnosis of depression via self-reporting. Moreover, risk factors, whose investigation this study ignored, may mediate the association between IC and race/color, such as living in a violent neighborhood and genetic risks. Furthermore, the theory of intersectionality considers factors which we failed to analyze, such as sexual orientation.

We believe that, although studies have reported that gait, verbal memory, and handgrip variables are closely associated with IC outcomes, research should investigate other domains, such as sleep, lower limb strength, balance, and blood biomarkers (hemoglobin and insulin-like growth factor 1 levels). Lastly, our cross-sectional experimental design prohibits us from establishing cause-and-effect relations. Future studies should evaluate IC sensory and psychosocial domains via medical diagnoses to reduce their risk of bias. Moreover, research should investigate the temporal relation between the lowest IC levels in historically marginalized groups and the incidence of disability in those populations.

The racial and gender disparities in IC we observed reinforce the need for public health policies to guarantee equality in the aging process. Promoting greater access to quality health care requires understanding how race/color disparities can contribute to health inequities and their consequences in different Brazilian regions.
